# The Serial Mediating Role of Cognitive Flexibility and Digital Literacy in the Relationship Between Metacognitive Awareness and Digital Well-Being

**DOI:** 10.3390/ejihpe16090127

**Published:** 2026-08-27

**Authors:** Selman Çutuk, Zeynep Akkuş Çutuk, Rukiye Aydoğan, Jason Perez, Pablo Prieto-González

**Affiliations:** 1 Independent Researcher, Edirne 22030, Turkey; selmancutuk4@gmail.com; 2 Department of Guidance and Psychological Counseling, Department of Educational Sciences, Faculty of Education, Trakya University, Edirne 22030, Turkey; zeynepacutuk@trakya.edu.tr; 3 Division of Recreation, Department of Recreation, Faculty of Sport Sciences, Aydın Adnan Menderes University, Aydın 09010, Turkey; rukiye.aydogan@adu.edu.tr; 4 Preparatory Year Program, College of Sciences and Humanities, Prince Sultan University, Riyadh 11586, Saudi Arabia; jperez@psu.edu.sa; 5 Sport Sciences and Diagnostics Research Group, College of Sciences and Humanities, Prince Sultan University, Riyadh 11586, Saudi Arabia

**Keywords:** metacognition, cognitive flexibility, digital literacy, digital well-being, structural equation modeling

## Abstract

(1) Background: As university students increasingly engage with digital tools in their academic and daily lives, digital well-being has become an important issue. This study aimed to examine a comprehensive sequential mediation model in which cognitive flexibility and digital literacy explain the relationship between metacognitive awareness and digital well-being among university students. (2) Methods: This cross-sectional correlational study included 711 university students. Data were collected using the Metacognitive Awareness Scale, Cognitive Flexibility Inventory, Digital Literacy Scale, and Digital Well-Being Scale. The proposed model was tested using structural equation modeling, and indirect associations were examined using bootstrap analyses. (3) Results: The findings indicated that metacognitive awareness was positively associated with cognitive flexibility and digital literacy, whereas cognitive flexibility was positively associated with both digital literacy and digital well-being. Digital literacy was also positively associated with digital well-being. Bootstrap results supported significant indirect associations between metacognitive awareness and digital well-being through cognitive flexibility and digital literacy, including their sequential mediation. (4) Conclusions: These findings suggest that university students’ digital well-being can be better understood by considering metacognitive awareness, cognitive flexibility, and digital literacy together. Therefore, holistic approaches that focus on developing these three areas together may be beneficial in interventions aimed at supporting digital well-being.

## 1. Introduction

### 1.1. The New Dimension of Well-Being in the Digital Age: Digital Well-Being

Digital technologies have become deeply embedded in contemporary life, reshaping how individuals learn, communicate, work, and maintain social relationships. The expansion of online education, remote work, social media, and mobile applications has intensified individuals’ engagement with digital environments and positioned technology at the center of daily routines (Gowthami & Kumar, 2016; Sarwar & Soomro, 2013). This transformation has also altered the conditions under which psychological functioning and well-being are experienced. In the digital age, well-being is no longer determined solely by social, emotional, and academic factors, but also by the extent to which individuals effectively regulate and manage their interactions with digital technologies.

In this context, digital well-being has emerged as an important construct for understanding individuals’ capacity to maintain a healthy, balanced, and functional relationship with digital environments (Al-Mansoori et al., 2023; Büchi, 2024).

Digital well-being does not simply refer to reducing screen time or minimizing technology-related stress. Rather, it involves the ability to use digital tools in ways that support psychological well-being, social connectedness, self-regulation, and the pursuit of meaningful goals. Consistent with broader models of psychological well-being, digital well-being may include the capacity to maintain autonomy, purpose, self-actualization, and adaptive functioning in digitally mediated contexts (Ryff & Singer, 2008).

The Dynamic System Model of Digital Well-Being provides a useful theoretical perspective for understanding this process. According to this model, digital well-being depends on achieving a balance in which digital distractions and dysfunctions are minimized, while social connectedness, cognitive control, and meaningful digital engagement are maximized (Vanden Abeele, 2021). From this perspective, digital well-being depends not only on access to technology, but also on individuals’ ability to regulate attention, evaluate digital information, adapt to changing digital demands, and engage with technology in a conscious and competent manner. Therefore, digital well-being should be examined through cognitive and self-regulatory mechanisms that explain how individuals manage their digital experiences.

### 1.2. The Relationship Between Metacognitive Awareness and Cognitive Flexibility

The rapid development of digital technologies has profoundly transformed not only individuals’ daily life practices but also their cognitive processes and behavioral patterns. Digitalization is expected to reshape not only physical labor but also cognitive and decision-making processes, requiring individuals to function in digital environments with greater flexibility, awareness, and strategic competence (Frey & Osborne, 2015). In this context, managing digital information flow, sustaining attention, and regulating engagement with digital tools require not only technical proficiency but also advanced cognitive capacities.

Metacognitive awareness refers to individuals’ awareness and regulation of their own cognitive processes, including the ability to monitor, evaluate, and adjust thinking strategies (Flavell, 1979; Schraw & Dennison, 1994; Schraw & Moshman, 1995). In digital information environments, the effective use of skills such as critical thinking, information evaluation, attention regulation, and content filtering depends largely on the deployment of metacognitive strategies. Recent studies suggest that metacognitive awareness is positively associated with managing cognitive load in digital contexts, reducing digital distractions, and building resilience against digital stress (Byzova et al., 2019; Ölmez & Sümen, 2025).

Current theoretical models conceptualize metacognition as a high-level self-regulatory capacity that coordinates cognitive, emotional, and motivational systems and supports individuals’ conscious efforts to achieve optimal functioning (Mitsea et al., 2024). In this regard, metacognitive awareness may be closely linked to cognitive flexibility. By enabling individuals to monitor, evaluate, and regulate their thinking, metacognitive awareness may help them shift perspectives, revise ineffective strategies, and adapt to changing demands. Consistent with this view, previous research has shown that metacognitive awareness is positively associated with cognitive flexibility through mechanisms involving monitoring, evaluation, and regulation of thoughts (Musullulu et al., 2025; Preiss, 2022).

**H1.**  *Metacognitive awareness is positively associated with cognitive flexibility.*

### 1.3. The Mediating Role of Cognitive Flexibility

Cognitive flexibility is defined as an individual’s capacity to adapt to changing environmental conditions, evaluate various perspectives, and develop alternative solutions (Bernardo & Presbitero, 2018; Ionescu, 2012). This capacity plays a critical role in the execution of complex cognitive processes, particularly in managing multiple tasks and developing strategies appropriate to dynamic demands (Johnco et al., 2013). In digital environments, such demands are especially salient because individuals frequently encounter rapidly changing information, multiple platforms, competing sources, and continuous interruptions.

Cognitive flexibility involves an individual’s ability to evaluate problem situations from different perspectives, switch between various cognitive strategies, and question the appropriateness of these strategies according to the situation (Canas et al., 2006). According to the model proposed by Martin and Anderson (1998), cognitive flexibility consists of three core components: awareness of available alternatives, willingness to exhibit flexible behavior, and perceived self-efficacy in behaving flexibly. These components are highly relevant to digital well-being because individuals who can evaluate alternatives and adapt their strategies may be better equipped to manage digital overload, avoid rigid technology-use patterns, and engage with digital tools in more balanced ways.

Cognitively flexible individuals can carefully analyze new situations and restructure their behavior accordingly; conversely, rigid thinking may trap individuals in fixed and maladaptive behavior patterns (Bernardo & Presbitero, 2018; Canas et al., 2006). In digital environments, such rigidity may weaken individuals’ capacity to cope with maladaptive digital behaviors, including problematic social media use and digital addiction tendencies. Indeed, recent findings indicate that higher levels of cognitive flexibility may have a protective role in coping with digital addiction behaviors (Çekiç et al., 2024). Therefore, cognitive flexibility may function as a mediating mechanism in the relationship between metacognitive awareness and digital well-being.

**H2.**  *Cognitive flexibility mediates the association between metacognitive awareness and digital well-being.*

### 1.4. The Instrumental Role of Digital Literacy

Digital literacy refers to individuals’ ability to access, evaluate, interpret, integrate, and produce information using digital technologies effectively and responsibly (Alkali & Amichai-Hamburger, 2004; Eshet, 2004; Julien, 2018). This concept is not limited to technical proficiency; rather, it includes the ability to interpret, make sense of, and produce diverse types of information in digital environments. The rapid pace of digital transformation has made digital literacy a key determinant of individuals’ learning behaviors. In this context, digital literacy is viewed not merely as a technological skill but also as an essential component of digital innovation and learner-centered pedagogical approaches (Audrin & Audrin, 2022; Eshet, 2004; Ng, 2012).

From an educational standpoint, digital literacy enhances students’ academic functioning by improving their ability to access, evaluate, and process information while also fostering critical thinking skills (Huang, 2024; Isrokatun et al., 2022; Meyers et al., 2013; Osterman, 2012; Tarsidi et al., 2023). Digital literacy may also be particularly relevant for digital well-being because it enables individuals to use technology in more conscious, safe, and balanced ways. A recent systematic review showed that literacy skills are strongly related to digital well-being among school-age students and emphasized the importance of integrating critical thinking with technical skills to support healthy digital development (Arkan & Bal, 2025). Individuals with stronger digital literacy skills may be better able to evaluate online information critically, regulate their digital engagement, manage online risks, and use digital tools for meaningful academic and social purposes.

Metacognitive awareness may also be directly associated with digital literacy. Individuals who monitor, evaluate, and regulate their cognitive processes may be better able to identify gaps in their digital knowledge, assess the credibility of online information, plan digital information searches, and revise ineffective strategies when interacting with digital technologies. Previous research provides conceptual support for this relationship by indicating that metacognitive strategies facilitate the monitoring and regulation of digital learning processes and promote more strategic engagement with digital environments (Lara Nieto-Márquez et al., 2020; Lavrysh et al., 2023; Yılmaz, 2016). Accordingly, a direct positive association between metacognitive awareness and digital literacy was hypothesized in addition to the indirect pathway through cognitive flexibility.

**H3.**  *Metacognitive awareness is positively associated with digital literacy.*

Cognitive flexibility is an important predictor of digital literacy in tasks such as searching for, analyzing, and integrating digital information (Caton et al., 2022; Tabullo et al., 2024). Individuals with high cognitive flexibility can more easily adapt to changing digital environments, learn new technologies, and develop effective strategies. These characteristics contribute to individuals’ ability to use digital tools effectively, critically, and consciously (Drigas et al., 2023). Therefore, digital literacy may function as a mediating mechanism in the relationship between cognitive flexibility and digital well-being.

**H4.**  *Digital literacy mediates the association between cognitive flexibility and digital well-being.*

### 1.5. Sequential Mediation Model: Metacognition → Cognitive Flexibility → Digital Literacy → Digital Well-Being

Although previous studies have examined the association between metacognitive awareness and general well-being (Chatterjee et al., 2021; Heshmati, 2018; Kiaei & Reio, 2014), less is known about how metacognitive awareness is associated with digital well-being. Existing research has generally focused on direct associations or single mediating mechanisms, such as mindfulness, emotional processes, or general self-regulatory capacities. However, digital well-being in contemporary educational contexts requires not only awareness of one’s thinking processes, but also the ability to adapt flexibly to changing digital demands and to use digital technologies critically, safely, and effectively.

Recent international evidence supports the need to examine digital well-being through cognitive and digital competence-based mechanisms. Studies from diverse educational contexts indicate that students’ digital functioning is shaped by digital literacy, digital cognitive load, and technology-related learning demands (Chen et al., 2025; Ibrahim et al., 2025; Zhou et al., 2025). These findings suggest that digital well-being cannot be fully explained through general well-being models alone; rather, it should be examined through cognitive adaptability and digital competence processes that reflect the demands of contemporary higher education.

Building on this perspective, the pathway from metacognitive awareness to digital well-being may involve both cognitive and digital mechanisms. Metacognitive awareness may support cognitive flexibility by enabling students to monitor and adjust their thinking strategies. Cognitive flexibility may then facilitate more adaptive engagement with complex digital environments, thereby strengthening digital literacy. Digital literacy, in turn, may contribute to digital well-being by helping students manage digital technologies in a more conscious, balanced, and responsible manner. However, studies that examine these mechanisms together within a sequential model remain limited (Șuşnea et al., 2024).

In the proposed model, cognitive flexibility was positioned before digital literacy because it represents a broad adaptive cognitive capacity that enables individuals to shift perspectives, respond to changing conditions, and revise ineffective strategies. Digital literacy, by contrast, reflects a more context-specific competence involving the critical, effective, and responsible use of digital technologies. Within this framework, cognitive flexibility was expected to support digital literacy by facilitating adaptation to new digital platforms, evaluation of alternative information sources, and modification of digital strategies when existing approaches prove ineffective (Caton et al., 2022; Tabullo et al., 2024).

Digital well-being was conceptualized as the distal outcome because it reflects the overall quality, balance, and adaptability of individuals’ functioning in digital environments. Metacognitive awareness, cognitive flexibility, and digital literacy were positioned as cognitive and competence-related resources associated with this outcome. However, because the study employed a cross-sectional design, the proposed ordering should be understood as theoretically specified rather than as evidence of temporal or causal precedence.

The present study addresses this theoretical and empirical gap by proposing a sequential mediation model in which cognitive flexibility and digital literacy explain the association between metacognitive awareness and digital well-being. Specifically, the study tests whether metacognitive awareness is associated with digital well-being through the sequential pathway of cognitive flexibility and digital literacy. By integrating metacognitive awareness, cognitive flexibility, and digital literacy into a single structural model, this study aims to clarify how higher-order self-regulatory awareness may be linked to students’ digital well-being through cognitive adaptability and digital competence.

Accordingly, the following research question guides the study:

Research Question. How are metacognitive awareness, cognitive flexibility, digital literacy, and digital well-being associated within a sequential cognitive-digital process model?

**H5.**  *Cognitive flexibility and digital literacy sequentially mediate the association between metacognitive awareness and digital well-being.*

## 2. Materials and Methods

### 2.1. Participants

This study was conducted during the spring semester of the 2024–2025 academic year with undergraduate students enrolled at two universities located in western Turkey. A convenience sampling approach was adopted in selecting the research group. Students who were present in the selected classes during the data-collection period were informed about the purpose and voluntary nature of the study and invited to participate. The inclusion criteria were being 18 years of age or older, having active undergraduate student status, and volunteering to participate in the study. Students who did not complete the questionnaire or who did not meet the inclusion criteria were excluded.

An a priori power analysis was conducted using G*Power 3.1 to determine the minimum sample size required for the study. Assuming a medium effect size (f^2^ = 0.15), α = 0.05, power = 0.95, and three predictors, the analysis indicated that at least 119 participants were required. The present study included 711 participants, confirming that the reached sample size was adequate to test the hypothesized serial mediation model. In addition, the sample size exceeded the minimum requirement suggested by Tabachnick and Fidell (2013) formula (*N* ≥ 50 + 8 m). In the present model, the most complex regression equation predicting digital well-being included three predictors: metacognitive awareness, cognitive flexibility, and digital literacy (m = 3). Accordingly, the minimum required sample size was 74 participants. Thus, the final sample size of 711 was well above both the GPower estimate and the Tabachnick and Fidell (2013) criterion.

A total of 720 questionnaires were administered. Nine questionnaires were excluded because of incomplete or invalid responses. The final sample comprised 711 participants, yielding a valid response rate of 98.75%. Of the participants, 58.1% were female (*n* = 413), and 41.9% were male (*n* = 298). Participants’ ages ranged from 18 to 48 years (M = 21.54, SD = 3.65). The sample consisted of first- to fourth-year undergraduate students, most of whom reported middle socioeconomic status.

Ethical approval was obtained from the Trakya University Social and Human Sciences Research Ethics Committee (Decision No. 2025.05.09), and institutional permission was obtained from both universities. Data were collected in classroom settings during class hours between May 10 and 15 June 2025. Before data collection, all participants provided written informed consent. Participant anonymity was maintained throughout the study, and all procedures were conducted in accordance with the ethical principles of the Declaration of Helsinki.

### 2.2. Measures

#### 2.2.1. Digital Well-Being Scale (DWBS)

The scale was developed by Arslankara et al. (2022). The scale consists of 12 items grouped into three subdimensions based on factor-analytic procedures: Digital Satisfaction, Safe and Responsible Behavior, and Digital Well-Being. Items are rated on a 5-point Likert-type scale ranging from 1 (Does not reflect at all) to 5 (Reflects completely). Total scores range from 12 to 60, with higher scores indicating greater well-being in digital environments. Regarding the reliability of the scale, the Cronbach’s alpha coefficient for the overall scale was reported as 0.87, the Guttman split-half coefficient as 0.78, and the Spearman–Brown coefficient also as 0.78.

#### 2.2.2. Metacognitive Awareness Scale (MAS)

The MAS was developed by Durdukoca and Arıbaş (2019). The scale consists of 18 items rated on a 5-point Likert-type scale and comprises three subdimensions: Personal Awareness, Organizational Awareness, and Judgmental Awareness. Higher scores indicate greater metacognitive awareness. Internal consistency was assessed using Cronbach’s alpha, with coefficients of 0.79, 0.72, and 0.62 for Personal Awareness, Organizational Awareness, and Judgmental Awareness, respectively. In addition, test–retest reliability over a four-week interval yielded correlation coefficients above 0.70 across all dimensions, indicating acceptable temporal stability.

#### 2.2.3. Cognitive Flexibility Inventory (CFI)

The CFI developed by Dennis and Vander Wal (2010) was adapted into Turkish by Gülüm and Dağ (2020). The inventory comprises 20 items and includes two subscales: Alternatives and Control. Participants rated the items on a 5-point Likert-type scale ranging from 1 (not at all appropriate) to 5 (completely appropriate). Internal consistency coefficients for the Turkish version were reported as 0.90 for the overall inventory, 0.89 for the Alternatives subscale, and 0.85 for the Control subscale. In addition, test–retest reliability over a two-week interval yielded correlation coefficients ranging from 0.35 to 0.50.

#### 2.2.4. Digital Literacy Scale (DLS)

The DLS developed by Ng (2012) was adapted into Turkish by Üstündağ et al. (2017). The scale consists of 10 items and demonstrates a unidimensional structure. Participants respond to the items using a 5-point Likert scale (1 = Strongly disagree, 5 = Strongly agree). The Cronbach’s alpha coefficient obtained in the adaptation study was 0.86, indicating a high level of internal consistency and reliability.

### 2.3. Data Analysis

Data analyses were performed using IBM SPSS Statistics (Version 25.0) and AMOS (Version 26) software packages. Descriptive statistics and Pearson correlation analyses were first conducted to summarize the study variables and examine their bivariate associations. Before testing the structural equation model, the relevant assumptions were evaluated. Univariate normality was assessed using skewness and kurtosis values, all of which fell within the conventional ±1 range, supporting the assumption of univariate normality. Additionally, the multicollinearity assumption was tested using VIF and Tolerance values. The diagnostic results showed that tolerance values ranged from 0.703 to 0.810, whereas VIF values ranged from 1.23 to 1.42. These values indicated no evidence of multicollinearity among the study variables, based on commonly accepted thresholds (VIF < 5, tolerance > 0.20).

After the assumption checks, a two-stage structural equation modeling (SEM) approach was used to examine whether cognitive flexibility and digital literacy sequentially mediated the association between metacognitive awareness and digital well-being. First, the measurement model was tested to evaluate the factor structure of the four latent constructs. Then, the structural model was tested to examine the hypothesized direct and indirect paths among the variables. To determine whether metacognitive awareness retained a direct association with digital well-being after cognitive flexibility and digital literacy were included as mediators, two nested structural models were compared. In the fully mediated model, no direct path was specified from metacognitive awareness to digital well-being, whereas this path was added in the partial mediation model. Model comparison was based on the chi-square difference test, the Akaike Information Criterion (AIC), the Expected Cross-Validation Index (ECVI), the statistical significance of the added direct path, and model parsimony. The more parsimonious model was retained when adding the direct path did not significantly improve model fit and the path coefficient was not statistically significant. In this comparison, full mediation referred specifically to the absence of the direct path from metacognitive awareness to digital well-being; the a priori path from metacognitive awareness to digital literacy was included in both models.

Model fit was evaluated using established goodness-of-fit indices. The following criteria were used to indicate acceptable model fit: χ^2^/df ≤ 5, CFI ≥ 0.90, NFI ≥ 0.90, TLI ≥ 0.90, IFI ≥ 0.90, GFI ≥ 0.85, RMSEA ≤ 0.08, and SRMR ≤ 0.08 (Hu & Bentler, 1999; Marcoulides & Schumacker, 2001; Schermelleh-Engel et al., 2003). These threshold values were selected considering the sample size of the current study (N = 711) and the complexity of the model (a moderately complex structure including four latent variables and ten observed variables). Due to the increased sensitivity of the χ^2^ statistic in large samples, the χ^2^/df ≤ 5 standard is widely recommended in the literature (Kline, 2023). Similarly, CFI, TLI, and RMSEA thresholds are also considered sufficient for indicating good fit in SEM studies with large samples (Hu & Bentler, 1999; Schermelleh-Engel et al., 2003). Model parameters were estimated using the maximum likelihood method. All standardized factor loadings and structural path coefficients were reported with 95% bias-corrected bootstrap confidence intervals based on 10,000 resamples. Standardized indirect effects were reported as effect-size estimates.

The Digital Literacy Scale was the only measure subjected to item parceling because it was conceptualized as unidimensional, consisted of 10 items, and did not include theoretically defined subscales. In contrast, Metacognitive Awareness, Cognitive Flexibility, and Digital Well-Being were represented by their established subscale scores, which served as theoretically meaningful indicators of the corresponding latent constructs. Parceling these multidimensional measures could have obscured their established internal structures. Item parceling was used to reduce the number of observed indicators and obtain a more parsimonious representation of the Digital Literacy construct in the SEM analysis (Little et al., 2002; Matsunaga, 2008). Parcels were created based on the corrected item-total correlations of the items, following the item-to-construct balancing approach. Accordingly, the items were distributed into two balanced parcels. Parcel 1 consisted of Items 1, 4, 6, 8, and 9, whereas Parcel 2 consisted of Items 2, 3, 5, 7, and 10. The mean scores of the items within each parcel were calculated, and the resulting parcel scores were included in the model as observed indicators of the latent Digital Literacy variable.

#### Common Method Bias Test

To assess whether common method bias posed a potential threat to the data, two diagnostic procedures were conducted. First, all observed items were examined using Harman’s single-factor test with an unrotated principal axis factoring approach (Podsakoff et al., 2003; Podsakoff et al., 2012). The results showed that the first factor accounted for 28.11% of the total variance. Because this value was below the conventional 50% threshold, the findings suggested that common method bias was unlikely to be a serious concern in the present study (Eichhorn, 2014). As an additional diagnostic check, the intercorrelations among the study variables were examined. The highest correlation among the variables was r = 0.63, which was well below the 0.90 threshold that may indicate discriminant validity concerns (Bagozzi et al., 1991). Taken together, these diagnostic indicators suggest that common method bias was unlikely to pose a substantial threat to the interpretation of the findings.

## 3. Results

### 3.1. Descriptive Statistics

Table 1 presents the descriptive statistics, internal consistency coefficients (Cronbach’s α and McDonald’s ω), skewness and kurtosis values, and the correlation matrix. The bivariate correlation analyses showed that metacognitive awareness was positively correlated with digital literacy (r = 0.63, *p* < 0.001), digital well-being (r = 0.46, *p* < 0.001), and cognitive flexibility (r = 0.53, *p* < 0.001). Digital literacy was also positively correlated with digital well-being (r = 0.49, *p* < 0.001) and cognitive flexibility (r = 0.45, *p* < 0.001). In addition, digital well-being was positively correlated with cognitive flexibility (r = 0.33, *p* < 0.001). The internal consistency coefficients were acceptable to high across the study variables (Cronbach’s α = 0.75–0.90; McDonald’s ω = 0.78–0.94). Skewness and kurtosis values fell within the conventional ±1 range, supporting the assumption of univariate normality.

Convergent and discriminant validity were examined for the measurement model. Table 2 presents the standardized factor loadings, their 95% bias-corrected bootstrap confidence intervals, indicator reliabilities, residual variances, composite reliability (CR), and average variance extracted (AVE) values. All freely estimated factor loadings were statistically significant (*p* < 0.001), and none of the bootstrap confidence intervals included zero. However, the magnitude of the loadings varied across constructs. The indicators of Metacognitive Awareness showed relatively strong loadings, ranging from 0.824 to 0.887, and the two Digital Literacy parcels loaded at 0.831 and 0.876. In contrast, the loadings for Cognitive Flexibility were 0.419 and 0.623, whereas those for Digital Well-Being ranged from 0.368 to 0.577.

The CR and AVE estimates indicated adequate convergent validity for Metacognitive Awareness (CR = 0.886, AVE = 0.722) and Digital Literacy (CR = 0.843, AVE = 0.729). By contrast, the corresponding estimates for Cognitive Flexibility (CR = 0.431, AVE = 0.282) and Digital Well-Being (CR = 0.461, AVE = 0.228) were below conventional benchmarks, indicating limited convergence among the subscale indicators used to represent these constructs. The discrepancy between the acceptable Cronbach’s alpha and McDonald’s omega coefficients and the lower CR and AVE values reflects differences in the level of measurement. Alpha and omega were calculated from the full sets of individual scale items, whereas CR and AVE were derived from the subscale and parcel indicators specified in the structural equation model. Thus, satisfactory item-level internal consistency did not necessarily imply strong convergence among the broader subscale indicators.

Indicators with comparatively low loadings were retained because the measurement specification was theory-driven and followed the established subscale structures of the original instruments. These indicators represented conceptually important dimensions of their respective constructs, and their removal would have substantially reduced construct coverage. In particular, excluding the Control subscale would have reduced Cognitive Flexibility to the Alternatives dimension alone, while removing one or more Digital Well-Being subscales would have narrowed the conceptual scope of that construct. Nevertheless, the low factor loadings and convergent validity estimates suggest that Cognitive Flexibility and Digital Well-Being were represented with lower measurement precision than Metacognitive Awareness and Digital Literacy. Accordingly, the direct and indirect associations involving these constructs should be interpreted cautiously, as measurement error may have affected the magnitude and precision of the estimated structural effects.

Discriminant validity was assessed using the Heterotrait–Monotrait ratio. All HTMT values were below 0.85, supporting the empirical distinctiveness of the four constructs. Taken together, the findings support discriminant validity across the measurement model, whereas convergent validity was established for Metacognitive Awareness and Digital Literacy but remained limited for Cognitive Flexibility and Digital Well-Being.

### 3.2. Structural Equation Modeling (SEM)

In the first stage of the analysis, a measurement model was tested to examine whether the observed variables adequately represented the latent constructs. The model included four latent variables—metacognitive awareness, cognitive flexibility, digital literacy, and digital well-being—represented by ten observed indicators. All freely estimated factor loadings were statistically significant (*p* < 0.001) and ranged from 0.368 to 0.887. However, several loading magnitudes were comparatively low, particularly for Cognitive Flexibility and Digital Well-Being, and were therefore interpreted in conjunction with the CR and AVE findings reported in Table 2. The measurement model demonstrated a good fit to the data: χ^2^ (29) = 87.669, χ^2^/df = 3.02, *p* < 0.001; GFI = 0.976; AGFI = 0.955; CFI = 0.980; NFI = 0.970; IFI = 0.980; SRMR = 0.028; RMSEA = 0.053, 90% CI [0.041, 0.067].

After evaluating the measurement model, two structural models were tested to examine a serial mediation model in which metacognitive awareness is associated with digital well-being through cognitive flexibility and subsequently digital literacy. In the first model (full mediation), no direct path was specified between metacognitive awareness and digital well-being; only indirect paths through the mediators were examined. This model also demonstrated a good fit: χ^2^ (30) = 88.677, χ^2^/df = 2.96, *p* < 0.001; GFI = 0.976; AGFI = 0.956; CFI = 0.980; NFI = 0.970; IFI = 0.980; SRMR = 0.028; RMSEA = 0.053, 90% CI [0.040, 0.065]; AIC = 138.677; ECVI = 0.196.

In the second model (partial mediation), a direct path from metacognitive awareness to digital well-being was added. The partially mediated model also showed good fit: χ^2^ (29) = 87.669, χ^2^/df = 3.02, *p* < 0.001; GFI = 0.976; AGFI = 0.955; CFI = 0.980; NFI = 0.970; IFI = 0.980; SRMR = 0.028; RMSEA = 0.053, 90% CI [0.041, 0.067]; AIC = 139.669; ECVI = 0.198. However, the added direct path was not statistically significant (β = 0.19, *p* > 0.05). A chi-square difference test revealed that adding this path did not significantly improve model fit (Δχ^2^ = 1.01, df = 1, *p* > 0.05). Therefore, the fully mediated model—with lower AIC and ECVI values—was retained as the final model.

**Figure 1:**
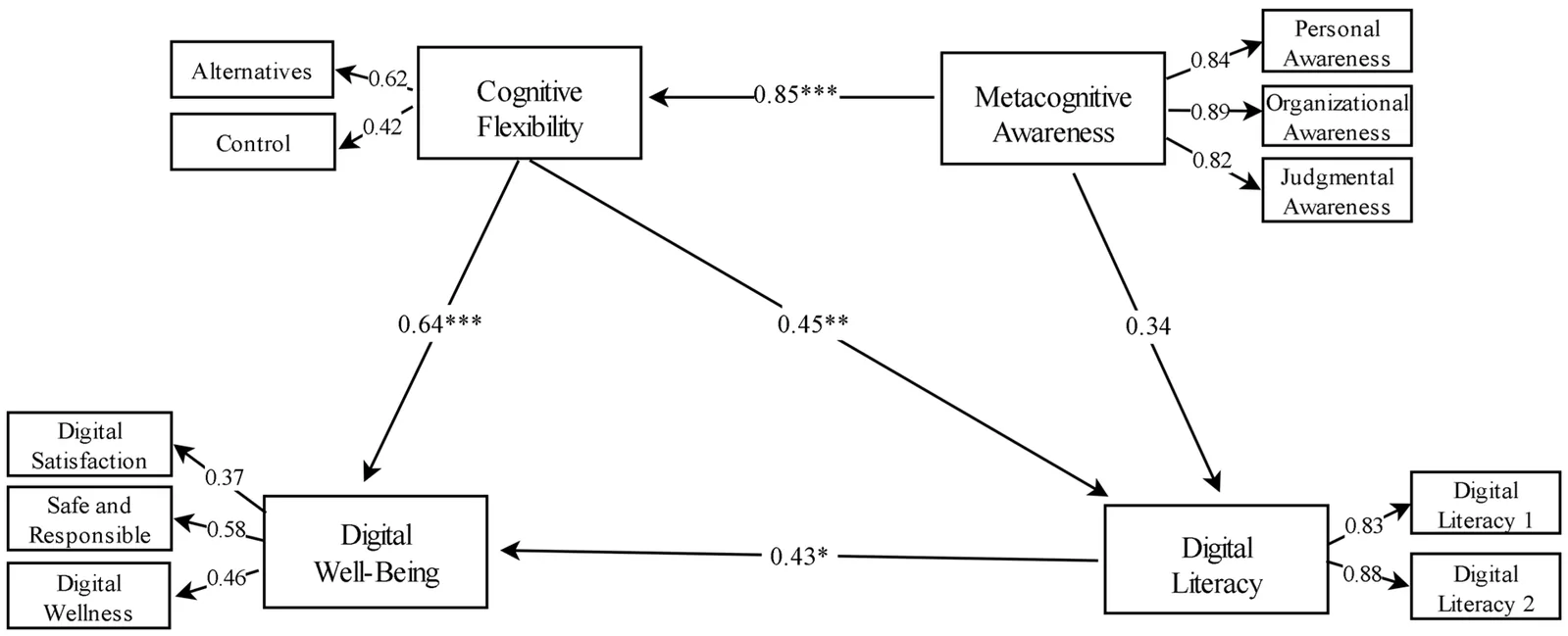
Standardized path coefficients for the retained serial mediation model. Note. Values shown on the measurement and structural paths are standardized estimates. The retained full mediation model excludes the direct path from Metacognitive Awareness to Digital Well-Being. The path from Metacognitive Awareness to Digital Literacy was specified a priori as a path to the second mediator and was included in both the full and partial mediation models. This path was not statistically significant in the bias-corrected bootstrap analysis. Bias-corrected bootstrap confidence intervals and significance levels for the structural coefficients are reported in Table 3, and the hypothesized specific indirect effects are reported in Table 4. * *p* < 0.05, ** *p* < 0.01, *** *p* < 0.001.

The standardized structural coefficients and their 95% bias-corrected bootstrap confidence intervals are presented in Table 3, while the retained structural model is illustrated in Figure 1.

**Table 3:** Standardized Structural Coefficients and Bias-Corrected Bootstrap Confidence Intervals.

Structural Path	β	95% BC CI	*p*
Metacognitive Awareness → Cognitive Flexibility	0.849	[0.773, 0.919]	<0.001
Cognitive Flexibility → Digital Literacy	0.445	[0.139, 0.953]	0.004
Metacognitive Awareness → Digital Literacy	0.341	[−0.153, 0.616]	0.133
Digital Literacy → Digital Well-Being	0.434	[0.130, 0.661]	0.011
Cognitive Flexibility → Digital Well-Being	0.644	[0.428, 0.915]	<0.001

Note. β = standardized structural coefficient; BC CI = bias-corrected bootstrap confidence interval. Confidence intervals were based on 10,000 bootstrap resamples.

**Table 4:** Specific Indirect Effects and Standardized Effect-Size Estimates.

Specific Indirect Pathway	B	95% BC CI	*p*	Standardized Indirect Effect
MA → CF → DL → DWB	0.048	[0.020, 0.112]	0.004	0.164
MA → CF → DWB	0.160	[0.100, 0.242]	<0.001	0.547
CF → DL → DWB	0.105	[0.044, 0.240]	0.004	0.193

Note. B = unstandardized indirect effect; BC CI = bias-corrected bootstrap confidence interval. Standardized indirect effects were calculated as the products of the corresponding standardized structural coefficients and are reported as effect-size estimates. MA = Metacognitive Awareness; CF = Cognitive Flexibility; DL = Digital Literacy; DWB = Digital Well-Being.

Metacognitive awareness was significantly and positively associated with cognitive flexibility (β = 0.849, 95% BC CI [0.773, 0.919], *p* < 0.001). Cognitive flexibility was significantly and positively associated with digital literacy (β = 0.445, 95% BC CI [0.139, 0.953], *p* = 0.004) and digital well-being (β = 0.644, 95% BC CI [0.428, 0.915], *p* < 0.001). Digital literacy was also significantly and positively associated with digital well-being (β = 0.434, 95% BC CI [0.130, 0.661], *p* = 0.011). Although the coefficient from metacognitive awareness to digital literacy was positive, its bias-corrected confidence interval included zero (β = 0.341, 95% BC CI [−0.153, 0.616], *p* = 0.133). Therefore, this direct association was not supported by the bootstrap results and was interpreted cautiously.

Finally, the bootstrap analysis based on 10,000 resamples supported the hypothesized indirect effects. Metacognitive awareness had a significant sequential indirect effect on digital well-being through cognitive flexibility and digital literacy (B = 0.048, 95% BC CI [0.020, 0.112], standardized indirect effect = 0.164, *p* = 0.004). A significant indirect effect was also observed through cognitive flexibility alone (B = 0.160, 95% BC CI [0.100, 0.242], standardized indirect effect = 0.547, *p* < 0.001). In addition, digital literacy significantly mediated the association between cognitive flexibility and digital well-being (B = 0.105, 95% BC CI [0.044, 0.240], standardized indirect effect = 0.193, *p* = 0.004). The standardized indirect effects were reported as effect-size estimates.

## 4. Discussion

The findings supported most of the hypothesized structural and indirect associations. First, metacognitive awareness was positively associated with cognitive flexibility. Second, cognitive flexibility accounted for an indirect association between metacognitive awareness and digital well-being. Third, digital literacy accounted for an indirect association between cognitive flexibility and digital well-being. Finally, the association between metacognitive awareness and digital well-being was indirectly linked through cognitive flexibility and digital literacy in sequence. Bootstrap analyses confirmed that this serial indirect association was statistically significant. These findings highlight the importance of considering not only metacognitive processes but also individuals’ cognitive flexibility and digital competencies in understanding digital well-being.

### 4.1. Metacognitive Awareness and Cognitive Flexibility

This study found that metacognitive awareness was significantly and positively associated with cognitive flexibility. It functions as a regulatory mechanism in the creative thinking process, managing the focus and release of attention and facilitating transitions between the phases of productivity and discovery (Preiss, 2022). Awareness of one’s own thought processes, the ability to modify them when necessary, and the development of alternative strategies constitute the foundation of cognitive flexibility (İdawati et al., 2020). This skill also supports the ability to generate alternative solutions when previous strategies prove ineffective (Bampa et al., 2024).

Findings in the field of education indicate that the effective use of metacognitive knowledge is associated with better attainment of students’ learning goals (Yang & Li, 2023) and that integrating metacognitive strategies into learning processes may support responsibility-taking and cognitive flexibility skills (Aydın et al., 2022). However, metacognitive skills were found not to mediate the relationship between cognitive flexibility and academic achievement (GPA). This finding suggests that while both skills contribute to the regulation of students’ learning processes, they exert their effects through distinct mechanisms (Musullulu et al., 2025).

Metacognitive awareness and cognitive flexibility, while generally producing positive cognitive outcomes, can limit creativity, increase cognitive load, and lead to decision fatigue when they are excessive or occur at inappropriate cognitive stages (Preiss, 2022). In contrast, low cognitive flexibility may lead to rigidity, stress, and slower thinking tendencies in the learning process. These findings suggest that metacognitive awareness and cognitive flexibility are not unidirectional processes but operate in mutual interaction. While excessive flexibility can cause indecision in strategy selection, insufficient flexibility may hinder adaptation to new situations. Particularly in managing cognitive processes in digital environments, the balanced and context-sensitive use of both skills is essential for supporting individuals’ cognitive functioning and digital well-being (İdawati et al., 2020).

### 4.2. The Mediating Role of Cognitive Flexibility

Another important finding obtained in this study is that cognitive flexibility accounted for a significant indirect association in the relationship between metacognitive awareness and digital well-being. This result suggests that individuals’ ability to adapt to and solve problems in digital environments may be associated with higher levels of digital well-being. Indeed, previous research has also emphasized that cognitive flexibility is a critical competency for adapting to the changing conditions of the digital age and maintaining psychological well-being (Dağgöl, 2023; Fuchs et al., 2023). Furthermore, it has been reported that cognitive flexibility is associated with better digital task performance and innovation productivity, with this effect being particularly pronounced in the context of digital innovation (Liang et al., 2025).

However, it should also be noted that this relationship is context-sensitive. Under conditions such as high cognitive load, multitasking digital environments, and constant distractions, excessive use of cognitive flexibility can lead to decision fatigue, cognitive dissonance, and mental exhaustion in individuals (Hasan, 2024; Mahdavi et al., 2024). Research shows that attention control weakens in multitasking environments and that flexible cognitive transitions can reduce information processing efficiency (Ophir et al., 2009; Ralph et al., 2014). Similarly, excessive flexibility may cause indecision or cognitive conflict in strategy selection, while low flexibility may lead to rigid thinking patterns and difficulties in adaptation (Galyam & Le Grange, 2005; İdawati et al., 2020). Therefore, it can be said that cognitive flexibility may not produce direct positive outcomes in every situation, and its effect is largely determined by the individual’s self-regulation capacity, emotional resilience, and level of digital awareness.

### 4.3. The Mediating Role of Digital Literacy

This study found that digital literacy plays a significant mediating role in the relationship between cognitive flexibility and digital well-being. Cognitive flexibility, as a skill related to the adaptation of thoughts and behaviors in response to changing environmental conditions, may be an important correlate of digital literacy. This skill may facilitate individuals’ transition between different digital tasks and strategies, supporting the processes of information search, evaluation, and integration (Tabullo et al., 2024). It has been demonstrated that cognitive flexibility in educational settings may be associated with higher learning flow and academic performance through the mediating role of digital literacy (Caton et al., 2022; Lee & Kim, 2023).

Digital literacy is not only a cognitive skill, but also an important psychosocial competency related to an individual’s level of online resilience. Individuals with high digital literacy may be more effective at identifying online risks and coping with negative experiences (Vissenberg et al., 2022). Digital conflicts and information overload were found to be significant predictors of emotional exhaustion, explaining 41% of the variance in this stressor (Sevic et al., 2025). However, existing evidence suggests that this effect may be context-dependent. Al-Youzbaky et al. (2022) found that excessive exposure to information and social media fatigue negatively influence decision-making processes by increasing technostress and information anxiety. Similarly, Ren et al. (2025) emphasized that information retrieval self-efficacy plays a key role in the relationship between digital competence and digital stress, and that high levels of digital stress may constrain this mechanism. Moreover, a high digital load has been associated with psychological pressures such as technostress and burnout among students (Tafesse et al., 2024). These findings indicate that the influence of digital literacy on digital well-being is likely a multidimensional construct shaped by contextual factors and individual differences.

### 4.4. Sequential (Serial) Mediation

The present study finally indicated that metacognitive awareness was indirectly associated with digital well-being in a sequential manner through cognitive flexibility and digital literacy. This result suggests that higher metacognitive awareness may be linked to greater cognitive flexibility, which in turn may be associated with higher levels of digital literacy and digital well-being. Metacognitive processes are not limited to individual learning outcomes but have also been associated with higher-order cognitive functions such as decision-making, problem-solving, and social adaptation (Varshney & Barbey, 2021). Therefore, digital well-being should be considered within a broader cognitive and behavioral system context guided by metacognitive processes.

Empirical findings in the literature support these research results. Previous research has suggested that metacognitive strategies may support students’ abilities to analyze, evaluate, and restructure complex information, thereby providing greater cognitive flexibility in learning processes (Pereles et al., 2024). Similarly, individuals with higher cognitive flexibility have been found to achieve meaningful improvements in learning outcomes by effectively processing complex content in digital reading environments (Cáceres-González et al., 2025). Other studies have suggested that metacognitive strategies may enhance metacognitive knowledge and regulation skills in the context of digital narratives (Lavrysh et al., 2023), that feedback provided on online platforms increases students’ capacity to monitor and regulate their learning process (Lara Nieto-Márquez et al., 2020), and that metacognitive awareness in digital environments encourages strategic digital behavior (Yılmaz, 2016).

On the other hand, despite the positive associations of metacognitive awareness with cognitive flexibility and digital literacy, it should be noted that excessive focus on technology can lead to cognitive overload and negatively affect learning outcomes (Caton et al., 2022). This finding emphasizes that interventions aimed at supporting digital well-being should focus not only on enhancing cognitive skills but also on balancing cognitive load.

### 4.5. Limitations and Suggestions for Future Research

Several limitations should be considered when interpreting the findings of this study. First, the cross-sectional design precludes definitive causal inferences among the study variables. Although the proposed structural equation model was grounded in relevant theoretical assumptions, longitudinal and experimental studies are needed to clarify the temporal ordering and causal directions among metacognitive awareness, cognitive flexibility, digital literacy, and digital well-being.

Second, the data were collected using self-report measures, which may be susceptible to response biases, including common method variance and social desirability. Future studies could address this limitation by adopting multi-method designs that incorporate qualitative data, behavioral indicators of digital engagement, or external assessments such as teacher and peer reports.

Third, an additional limitation concerns the measurement properties of Cognitive Flexibility and Digital Well-Being. Although both instruments demonstrated acceptable item-level internal consistency, the Composite Reliability (CR) and Average Variance Extracted (AVE) estimates based on the subscale indicators used in the structural equation model were below conventional thresholds. The comparatively low standardized factor loadings indicate limited convergence among the subscales representing these latent constructs. Consequently, the direct and indirect associations involving Cognitive Flexibility and Digital Well-Being should be interpreted cautiously. The statistical significance of these associations does not eliminate the underlying measurement limitations, and the magnitude and precision of the estimated structural effects may have been affected by measurement error. Future studies should replicate the proposed model using alternative measurement specifications and instruments demonstrating stronger convergent validity.

Finally, the sample consisted exclusively of higher education students from a specific educational and cultural context, which may limit the generalizability of the findings to other age groups, educational levels, and cultural settings. Future research should therefore test the proposed model in more diverse samples and across different cultural contexts.

In terms of future research, although the findings are consistent with a linear model, it should be acknowledged that reciprocal or cyclical interactions may exist among the variables. For instance, higher levels of digital well-being may, over time, reinforce individuals’ cognitive flexibility and their ability to use digital environments in a more conscious, balanced, and strategic manner. Future studies are encouraged to test alternative models that incorporate such cyclical dynamics and to explore the mediating or moderating roles of emotional and motivational factors.

### 4.6. Conclusions and Implications

The findings of this study suggest that metacognitive awareness, cognitive flexibility, and digital literacy are complementary cognitive and behavioral components associated with digital well-being. Cognitive flexibility may facilitate information management in digital environments and may be linked to higher digital literacy and digital well-being.

At the educational level, the findings indicate that universities and digital learning platforms may benefit from integrating content that fosters metacognitive awareness and cognitive flexibility skills into their curricula. Course modules that equip students with problem-solving, self-regulation, and digital strategy skills may contribute to healthier and more productive digital learning experiences. Furthermore, considering that digital literacy extends beyond technical competencies to include broader dimensions such as digital ethics, information verification, online self-awareness, and attention management, classroom applications and micro-learning activities may be designed to address these areas.

At the psychoeducational and student-support levels, the findings may inform digital awareness workshops, digital stress management activities, and preventive programs aimed at supporting students’ digital well-being. Programs that encourage awareness of metacognitive strategies, flexible thinking, and responsible digital literacy practices may help students engage with digital environments more consciously and adaptively. However, given the non-clinical and cross-sectional nature of the sample, these implications should be interpreted cautiously. Future longitudinal and intervention-based studies are needed to determine whether strengthening these skills leads to improvements in digital well-being.

## Figures and Tables

**Table 1:** Descriptive statistics, reliabilities and correlations for the study variables.

	M	SD	Skewness	Kurtosis	α	ω	1	2	3	4
1. Metacognitive Awareness Scale (MAS)	62.72	10.66	0.24	0.20	0.84	0.93	-			
2. Digital Literacy Scale (DLS)	35.03	6.59	0.05	0.07	0.90	0.94	0.63 **			
3. Digital Well-Being Scale (DWBS)	40.95	6.13	0.63	0.85	0.75	0.78	0.46 **	0.49 **		
4. Cognitive Flexibility Inventory (CFI)	75.26	10.18	0.22	−0.61	0.82	0.84	0.53 **	0.45 **	0.33 **	-

Note. ** *p* < 0.001.

**Table 2:** Standardized Factor Loadings, Bias-Corrected Bootstrap Confidence Intervals, Indicator Reliability, Residual Variance, Composite Reliability, and Average Variance Extracted.

Construct	Indicator	Standardized Loading	95% BC CI	Indicator Reliability	Residual Variance	CR	AVE
Metacognitive Awareness	Personal Awareness	0.837	[0.802, 0.868]	0.701	0.299	0.886	0.722
	Organizational Awareness	0.887	[0.853, 0.912]	0.787	0.213		
	Judgmental Awareness	0.824	[0.787, 0.857]	0.679	0.321		
Cognitive Flexibility	Control	0.419	[0.348, 0.486]	0.176	0.824	0.431	0.282
	Alternatives	0.623	[0.522, 0.711]	0.388	0.612		
Digital Literacy	Digital literacy 1	0.831	[0.794, 0.863]	0.691	0.309	0.843	0.729
	Digital literacy 2	0.876	[0.838, 0.911]	0.767	0.233		
Digital Well-Being	Digital Satisfaction	0.368	[0.272, 0.463]	0.135	0.865	0.461	0.228
	Safe and Responsible Behavior	0.577	[0.498, 0.642]	0.333	0.667		
	Digital Well-Being	0.463	[0.376, 0.543]	0.214	0.786		

Note. CR = composite reliability; AVE = average variance extracted. Indicator reliability was calculated as the squared standardized factor loading ($\lambda^{2}$), and residual variance was calculated as $1-\lambda^{2}$. CR and AVE are construct-level estimates and are therefore reported once for each latent construct. All freely estimated factor loadings were statistically significant at p<0.001. BC CI = bias-corrected bootstrap confidence interval. Confidence intervals were based on 10,000 bootstrap resamples.

## Data Availability

The dataset containing the variables analyzed in the present manuscript is available from the corresponding author upon reasonable request.
